# Comparative toxicity of two neonicotinoid insecticides at environmentally relevant concentrations to telecoprid dung beetles

**DOI:** 10.1038/s41598-023-35262-w

**Published:** 2023-05-26

**Authors:** Michael C. Cavallaro, Michelle L. Hladik, Samantha Hittson, Greg Middleton, W. Wyatt Hoback

**Affiliations:** 1 Department of Entomology and Plant Pathology, Oklahoma State University, Stillwater, OK 74078 USA; 2 U.S. Geological Survey, California Water Science Center, Sacramento, CA 95819 USA

**Keywords:** Environmental impact, Entomology

## Abstract

Dung beetles (Coleoptera: Scarabaeinae) frequently traverse agricultural matrices in search of ephemeral dung resources and spend extended periods of time burrowing in soil. Neonicotinoids are among the most heavily applied and widely detected insecticides used in conventional agriculture with formulated products designed for row crop and livestock pest suppression. Here, we determined the comparative toxicity of two neonicotinoids (imidacloprid and thiamethoxam) on dung beetles, *Canthon spp.*, under two exposure profiles: direct topical application (acute) and sustained contact with treated-soil (chronic). Imidacloprid was significantly more toxic than thiamethoxam under each exposure scenario. Topical application LD50 values (95% CI) for imidacloprid and thiamethoxam were 19.1 (14.5–25.3) and 378.9 (200.3–716.5) ng/beetle, respectively. After the 10-day soil exposure, the measured percent mortality in the 3 and 9 µg/kg nominal imidacloprid treatments was 35 ± 7% and 39 ± 6%, respectively. Observed mortality in the 9 µg/kg imidacloprid treatment was significantly greater than the control (*p* = 0.04); however, the 3 µg/kg imidacloprid dose response may be biologically relevant (*p* = 0.07). Thiamethoxam treatments had similar mortality as the controls (*p* > 0.8). Environmentally relevant concentrations of imidacloprid measured in airborne particulate matter and non-target soils pose a potential risk to coprophagous scarabs.

## Introduction

Agricultural intensification continues to threaten non-target terrestrial insect communities^1^, where neonicotinoids are among the key drivers of decreased abundance in beneficial insects^2,3^. Broad-spectrum neonicotinoids are the most widely used class of insecticides worldwide, constituting 30% of all insecticide sales^4–6^. The insecticidal activity of neonicotinoids is mediated by agonist action on the nicotinic acetylcholine receptors (nAChR), where ingestion or cuticle penetration elicits lethal and sub-lethal effects^7^. Because of the shared physiology among insect taxa, target and non-target insects are equally susceptible to the effects of insecticide exposure, albeit with varying degrees of sensitivity^8^. Studies characterizing the fate and transport of neonicotinoids confirm their mobility in the environment, resulting in acute and chronic exposure to beneficial insects^9^. Krupke and Tooker^10^ estimate greater than 90% of neonicotinoid active ingredient (AI) mass applied to seeds could move off-site and persist in non-target environmental media. Landscape-level agricultural matrices (i.e., conventional row cropland and livestock feedlots) often contain conservation easements, rangeland, and native vegetative buffers^11–13^, potentially affecting nearby habitats designed to conserve biodiversity. Multiple routes of acute and chronic neonicotinoid exposure are well-defined^14^, and terrestrial insects are particularly at risk to incidental contact via airborne or soil-bound AIs.

Conventional agriculture operations emit air pollutants that contribute to the dissemination of neonicotinoids bound to particulate matter^15,16^. Neonicotinoid-treated seeds are abraded by pneumatic drillers during planting, which use seed lubricants (e.g., talc); seed lubricants mix with the released AI particles and a portion are expelled through the exhaust system as seed dust (reviewed by Nuyttens et al. ^17^). Concentrations of particulate matter (settled and airborne) can exceed topical contact toxicity thresholds for honeybees^18–20^. Recently, comparable concentrations of neonicotinoids were measured in windblown particulate matter from cattle feedlots^16^. Livestock pest suppression products include multiple topical, feed-through, and residual formulations that allow AI-bound particles to accumulate in feedlot facilities^21^. Moreover, several granular and spray fly bait products contain neonicotinoids^22^. Contaminated particles will disperse with wind and settle on surfaces near conventional row cropland and livestock feedlots, including vegetation, surface soil, and directly on insects^20–24^.

Seed treatment technology—representing the primary method of applied neonicotinoids in row crops—has increased overall chemical inputs to soils^5,6,25–27^. Recent inventories total 263 formulated seed treatment products, containing 56 AIs applied as single or multiple compounds^28^. As systemic insecticides, neonicotinoids are highly water-soluble, and once in soil, AIs are released from the seed treatments and dissolved in water during subsequent irrigation. Crops will uptake and incorporate AIs into tissues over time, protecting seedlings from early-stage arthropod herbivory^29^. However, most of the applied AI remains in the soil with the uptake from targeted crops ranging from 1.6 to 20%^30^. More recent investigations measure the percent of AI uptake in target plants to be 2–3%^31^. AI half-life in soil often exceeds planting intervals, and persistence is highest in dry soils with high organic matter, which are common in agricultural settings^32^. In some landscapes, these conditions will lead to the environmental loading of AIs in surrounding habitats and frequent detection beyond cultivated areas^12,32,33^.

Globally, coprophagous beetle communities in the subfamily Scarabaeinae (Coleoptera: Scarabaeidae) are being reshaped by anthropogenic stressors with most monitored populations in decline^34–39^. Credited as important bioindicators of ecosystem health^40^, dung beetles perform a variety of ecological functions, which differ among taxa^41^. Dung is an ephemeral resource, and its spatial reliability will differ from local- to landscape-levels^42^. To negotiate the spatiotemporal dynamics of dung availability, adult foraging activity and diel flight patterns (i.e., diurnal, nocturnal, and crepuscular) are species-specific and vary seasonally^42,43^. Once reached, dung-use and brood ball burial is broadly defined by three behavioral guilds: tunnellers (paracoprid), rollers (telecoprid), and dwellers (endocoprid); the depth and interaction with surface soils differs among guilds and beetle size^41,44,45^.

Numerous studies have reported the negative effects of insecticides measured in dung (primarily pyrethroids) on larvae and adult beetle abundance^46–50^. Considering the possibility of recurrent neonicotinoid exposure scenarios and the life history strategies of dung beetles, airborne active ingredients carried in seed dust or contaminated particulate matter from livestock feedlots and surface soils exposed to direct planting, nearby agricultural runoff, or dusting may also pose a substantial threat to beetle survival.

Here, we assessed two potential routes of exposure: direct contact via particulate matter (acute) and sustained contact with field-realistic surface soil concentrations (chronic). The neonicotinoid AIs imidacloprid and thiamethoxam were selected because of their widespread use in conventional agriculture (i.e., row crops and feed operations) and subsequent detection in the environment^4,5,16,32^. Telecoprid dung beetles, *Canthon spp*., represent one of the most abundant and diverse genera of Scarabaeinae at the study site^51^ and a behavioral guild potentially at greater risk of neonicotinoid exposure, i.e., vagile with dung burial in shallow nests beneath the pat. We hypothesized that imidacloprid and thiamethoxam will have measurable effects on *Canthon spp*. at environmentally relevant concentrations throughout each exposure profile with comparable responses between the two active ingredients.

## Materials and methods

### Insect collection

Adult *Canthon spp*. were collected from the Tallgrass Prairie Preserve in Pawhuska, Oklahoma, USA (36°50′27.6′′N, -96°26′24′′W), the largest intact tallgrass prairie remaining in the United States with no known previous use of neonicotinoids. Pitfall traps were baited with approximately 40 g of pig, *Sus scrofa*, dung and checked every 24 h. Captured beetles were transported back to the laboratory in moistened peat moss and sorted into separate plastic containers. During the sampling period, approximately 60% of captures were *Canthon spp*., and the most abundant species was *Canthon chalcites* Haldeman with a small fraction (< 5%) of *Canthon vigilans* LeConte. Beetles were collected from August to September 2022. Before tests, beetles were fed ad libitum on pig dung. Dung used for pitfall traps and during laboratory tests was acquired from the Oklahoma State University Swine Center. Pigs are dewormed annually each winter with a 1% ivermectin injection, nearly 9 months before dung collection and usage.

### Dose-response topical contact application

Technical grade imidacloprid and thiamethoxam were purchased from Sigma Aldrich (St. Louis, Missouri, USA) and used in all tests. Neonicotinoids were dissolved in analytical grade acetone in a nominal concentration series and were applied in 1 µL of acetone dispensed on the pronotum using a 10 µL microsyringe (Hamilton; Reno, Nevada). Imidacloprid concentrations were 0 (control), 6, 12, 24, 60, 120, 240 ng/µL, and thiamethoxam concentrations were 0 (control), 20, 40, 80, 160, 320, 640 ng/µL. Acute exposure concentrations were selected based on a series of range-finding tests, where test concentrations were initially selected from acute honeybee LD50 values (topical exposure) reported in Sánchez-Bayo and Goka^52^ and scaled accordingly. Each treatment was administered to 15 individuals per concentration for a total of 105 beetles per compound. Acetone-only treated individuals were included as controls. Beetles were weighed (mg), dosed, and placed in individual plastic cups. To prevent desiccation, beetles were supplied with a moist cotton ball; no food was provided. Mortality was assessed after 24 h. To assess mortality, beetles were placed on their backs and considered dead when they did not flip over after a 2 min observation period. Qualitative observations on beetle behavior and locomotor functions immediately after application were also recorded.

### Soil exposure bioassays

Soil was collected from a nearby grassland site with vegetated buffers. Soils were sieved to remove large debris, placed in aluminum pans, and oven-dried at 80 °C to a constant weight. Stock solutions of neonicotinoids were dissolved in carbon-filtered, reverse osmosis water. Volumes of stock solution were further diluted to achieve the target dose and percent soil moisture of 5%; this moisture level was targeted in previous laboratory-based beetle studies ^53^. Approximately, 2.5 kg of dried soil was used per treatment; using a gloved hand, neonicotinoid-spiked soils were thoroughly mixed. Control soils went through the same process with the addition of only water. Subsamples of soil from each treatment were collected, labelled, and frozen at day 10 for residue analyses and soil characterization (% sand/silt/clay, pH, % total organic carbon).

Each dose group was replicated four times and each replicate consisted of 9 beetles for a total of 180 beetles among all treatment groups and controls. Beetles were exposed for 10 days, representing a realistic soil exposure profile for this genus^54^. Experiments were kept in a climate-control laboratory space held at 21 ± 1 °C with a photoperiod of 16:8 (L:D). Replicate containers were provided with 45 g of pig dung on Day 0 and again on Day 6. Two target doses of imidacloprid and thiamethoxam were used: 3 and 9 µg/kg. Neonicotinoid concentrations were chosen based on environmentally relevant residue measurements from non-target soils found in the United States^12,55,56^. Qualitative observations of beetle activity were made throughout the test. After the 10-day exposure, mortality data were recorded. All beetles were frozen in − 20 °C immediately after test termination and stored until additional analysis. A subset of whole beetles from each replicate of the soil exposure bioassays was subjected to acetone rinses to quantify neonicotinoid residues on the cuticle of the beetles.

### Chemical analyses

#### Neonicotinoid-spiked acetone and residues on whole beetle cuticle

Topical imidacloprid and thiamethoxam acetone solutions were measured by directly injecting, or diluting and then injecting, 10 µL onto an Agilent Technologies (Santa Clara, California) 1260 infinity bio-inert high-performance liquid chromatograph coupled to a 6430 triple quadrupole mass spectrometer (LC–MS/MS). Dilutions were made using acetonitrile. Instrument details and parameters followed the previous work of Gross et al.^57^. The limit of detection (LOD) for both AIs was 0.0025 ng/µL.

Acetone rinses followed the same analytical methods as above. Frozen beetles stored at test termination were examined to ensure each beetle used for the neonicotinoid/metabolite residue analyses was intact. Individual beetles with missing limbs were not included. Due to the decreased number of intact beetles, three replicates were used per treatment. Nine beetles were rinsed in 9 mL of acetone for 10 min, i.e., 1 mL per beetle. The 1 mL acetone samples were concentrated to 0.2 mL in acetonitrile, and then 10 µL was injected onto the LC–MS/MS. The neonicotinoid metabolites analyzed included imidacloprid olefin, imidacloprid urea, imidacloprid, 5-hydroxy, and thiamethoxam degradate (CGA-355190)/thiamethoxam urea.

#### Soil analyses

Soil samples were extracted using a previously developed method^58^. Briefly, soils were freeze dried using a Labconco FreeZone 4.5 system. After freeze drying, 5 g samples were homogenized, spiked with a surrogate compound d_4_-imidacloprid (Cambridge Isotope, Andover, Massachusetts), at a concentration of 10 ng/g, and extracted using a CEM EDGE® Automated Extraction System (Matthews, North Carolina) with acetonitrile at 100 °C. The acetonitrile extracts were reduced to 0.5 mL under nitrogen gas, loaded onto a carbon solid phase extraction cartridge (Carboprep90 500 mg, 6 cc; Restek; Bellefonte, Pennsylvania) and eluted from the carbon cartridge with 10 mL of acetonitrile. The eluents were reduced to 0.2 mL under nitrogen gas and were spiked with an internal standard (d_3_-clothianidin; Cambridge Isotope). Samples were analyzed for imidacloprid, thiamethoxam, and metabolites using the LC–MS/MS method described above^57^. The LOD for each parent compound and metabolite analyzed was 0.1 µg/kg.

### Data analyses

Statistical analyses were conducted in R version 4.1.1^59^. Treated as proportion data, the *cbind* function was used to analyze the response variable (i.e., percent mortality) for the topical and soil neonicotinoid exposure scenarios. Acute, contact LDx values and their 95% confidence intervals were determined using generalized linear models (GLMs package stats) with a binomial distribution (logit link). Measured neonicotinoid concentrations were log-transformed and corrected for initial beetle weight (mg) to describe the dose (ng/beetle) received for each treatment. Threshold LDx concentrations were calculated using the *dose.p* function (package MASS;^60^). A similar model structure was used to determine the interaction effect between beetle weight and dose. Chronic, soil bioassay data were also analyzed using GLMs with a binomial distribution (logit link), where the fixed effects included the interaction terms: AI * dose. Interaction terms were selected after a comparison with likelihood ratio tests (LRT) between the additive and interaction models (ɑ = 0.05). We also coded five treatment levels, i.e., low and high dose per AI + control, and conducted pairwise comparisons (ɑ = 0.05) using Tukey tests with the *emmeans* function (package emmeans; ^61^). Results of posthoc comparisons are given on the log odds ratio, not the response scale^61^. Data were subjected to normality (package stats) and variance (package car;^62^) checks by Shapiro–Wilk and Levene's test, respectively, if required. Model fit distributions were further confirmed by a chi-square test based on residual deviance.

## Results

### Measured exposure concentrations

Mean measured neonicotinoid-spiked acetone concentrations were 88.7% and 102.8% of the target nominal doses for imidacloprid and thiamethoxam, respectively (Table 1). Acetone used for the method blank and control groups were below the LOD of 0.0025 ng/µL, i.e., no detection (ND), and the relative standard deviation among neonicotinoid dose groups was similar to the target concentration (Table 1). All calculated endpoints were based on measured concentrations and corrected for beetle weight. Soil bioassay samples were analyzed for imidacloprid and thiamethoxam residues at test termination on Day 10; results showed that mean measured soil concentrations were substantially lower than the target nominal dose. Mean imidacloprid soil concentrations were within 27.5% and 17.0% of the target nominal doses of 3 and 9 µg/kg, respectively. Mean thiamethoxam soil concentrations were 14.1% and 26.9% of the target nominal doses of 3 and 9 µg/kg, respectively (Table 1). Control soils were below the LOD of 0.1 µg/kg. Composite cuticle rinse samples among all dose groups and replicates were also below the LOD. Chemical analyses included imidacloprid and thiamethoxam metabolites.

**Table 1:** Measured neonicotinoid concentrations for each target nominal dose group by exposure scenario.

Route of exposure	Imidacloprid		Thiamethoxam	
	Nominal	Measured	Nominal	Measured
Topical contact (ng/µL)	0	ND^a^	0	ND
	6	5.0 ± 0.1	20	17.6 ± 0.9
	12	9.6 ± 0.2	40	35.6 ± 0.3
	24	19.3 ± 0.2	80	87.7 ± 2.7
	60	52.8 ± 1.0	160	194.7 ± 2.7
	120	113.3 ± 5.7	320	363.5 ± 4.3
	240	254.1 ± 3.4	632	599.2 ± 13.2
Soil exposure (µg/kg)^b^	0	ND	0	ND
	3	0.8 ± 0.1	3	0.4 ± 0.1
	9	1.5 ± 0.2	9	2.4 ± 0.2

^a^No detection =  < 0.02 µg/L in acetone; < 0.1 µg/kg in soil.

^b^Imidacloprid and thiamethoxam metabolites were not detected.

### Topical contact

**Figure 1:**
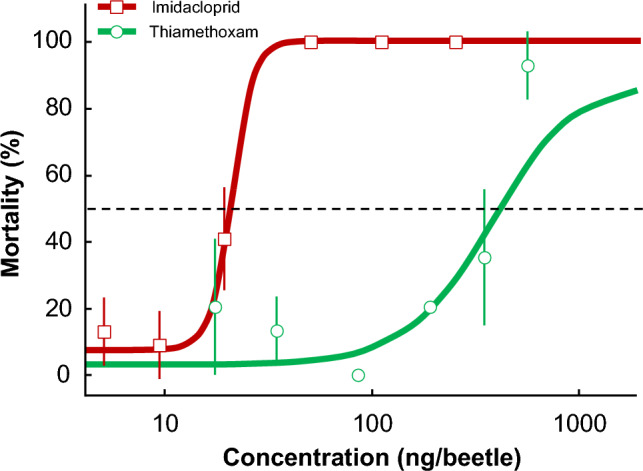
Dose–response curves (mean ± S.E.) of topically applied imidacloprid and thiamethoxam in *Canthon spp*. The dashed line indicates 50% mortality.

Imidacloprid was more toxic than thiamethoxam to *Canthon spp*. (Fig. 1). The LD50 values (95% CI) for imidacloprid and thiamethoxam were 19.1 (14.5–25.3) ng/beetle and 378.9 (200.3–716.5) ng/beetle, respectively. The LD90 value (95% CI) for imidacloprid was 43.3 (26.9–69.7) ng/beetle, whereas the LD90 value for thiamethoxam was greater than the highest measured dose in the concentration series, i.e., > 599.2 ng/beetle. Mortality for imidacloprid-exposed beetles was 100% for all concentrations above 52.8 ng/beetle, whereas mortality for the thiamethoxam-exposed beetles did not reach 100% mortality within the selected concentration series. Using the LD50 values, imidacloprid was nearly 20X more toxic than thiamethoxam. All control beetles topically exposed to acetone-only survived. Mean (± S.E.) beetle weight was 368 ± 6 mg with a range of 134–641 mg. The interaction between beetle weight and dose was not significant for either imidacloprid (β ± S.E. = − 1.78 ± 2.4, *p* = 0.46) or thiamethoxam (β ± S.E. =  − 0.94 ± 0.8, *p* = 0.25).

### Soil exposure

**Figure 2:**
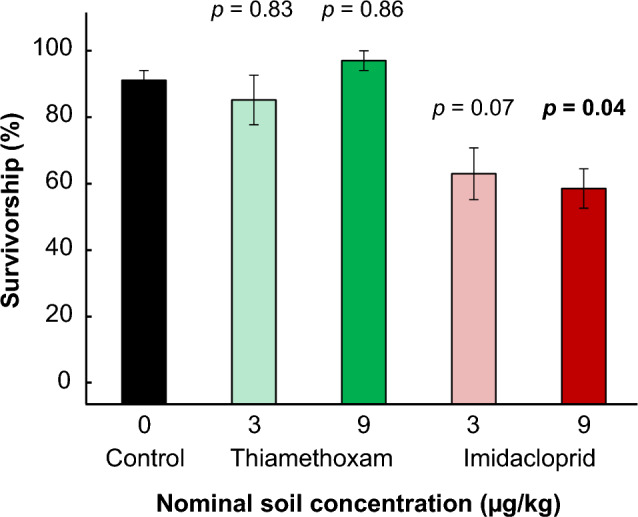
Percent survivorship (mean ± S.E.) of *Canthon spp*. from 10-day soil exposure to imidacloprid and thiamethoxam. P-values above treatments indicate whether the exposure was significantly different from the control as determined by post-hoc Tukey test (ɑ = 0.05).

Test soils were classified as sandy loam (i.e., 64% sand, 32% silt, 4% clay) with a pH of 8.4 and a total organic carbon of 1.6%. Beetles chronically exposed to imidacloprid experienced greater mortality than the control and thiamethoxam treatments (Fig. 2; β ± S.E. = 1.76 ± 0.8, *p* = 0.03). There was no significant difference between dose groups for each neonicotinoid tested (β ± S.E. = 0.32 ± 0.18, *p* = 0.08). Among the treatment groups, the 9 µg/kg imidacloprid dose was the only treatment with significantly greater mortality than the control group (β ± S.E. =  − 1.95 ± 0.7, *p* = 0.04). However, the 3 µg/kg imidacloprid dose response may still be environmentally relevant (β ± S.E. =  − 1.83 ± 0.7, *p* = 0.07). Thiamethoxam did not elicit a response at the nominal test concentrations with survivorship of 97 ± 3% at the highest dose. Accordingly, both the 3 µg/kg (β ± S.E. = 2.99 ± 1.1, *p* = 0.04) and 9 µg/kg (β ± S.E. = 3.10 ± 1.1, *p* = 0.03) imidacloprid treatments were significantly different from the 9 µg/kg thiamethoxam treatment. Mean control survivorship (± S.E.) was 92 ± 3% (Fig. 2).

## Discussion

### Comparative toxicity of imidacloprid and thiamethoxam

Numerous studies have documented the detrimental impacts of neonicotinoids on beneficial beetle taxa (reviewed by Pisa et al.^9^). Relative to the number of studies on the non-target effects of endectocide residues (e.g., ivermectin), there are fewer data on the ecotoxicity of insecticides to dung beetle populations^35,36^. Most of the research on dung beetle insecticide exposure has specifically focused on pyrethroids^46–50^ with some attention on the impacts of organophosphates, herbicides, and insect growth regulators^35,36,63^.

To our knowledge, no study has assessed the toxicity of neonicotinoids to dung beetles under acute and chronic exposure profiles. However, several formulated neonicotinoid products are designed to suppress defoliating and turf grass scarab beetle pests (Coleoptera: Scarabaeidae). In the context of pest suppression, previous studies have reported and reviewed the efficacy of neonicotinoids to the scarab subfamilies Dynastinae^64^, Melolonthinae^65,66^, and Rutelinae^65^, highlighting the known toxic effects of neonicotinoids to scarab beetles. Polavarapu et al.^67^ compared the efficacy of imidacloprid and thiamethoxam treatments on the root-feeding scarab *Anomala orientalis* Waterhouse. After two separate 21-day exposures, the results were inconclusive and variable, where third-instar larvae displayed higher mortality when exposed to thiamethoxam during the first trial and higher mortality when exposed to imidacloprid during the second trial^67^. Variables such as insecticide resistance, seasonality, or availability of AIs to larvae may have influenced differing responses. Another study examined mortality and behavioral response of two pest scarab species, the Japanese beetle *Popillia japonica* Newman and the northern masked chafer *Cyclocephala borealis* Arrow. Exposure to imidacloprid-treated turfgrass significantly lowered *P. japonica* second-instar larvae survivorship seven days post-application with no effect from the thiamethoxam treatment throughout the 31-day exposure. However, the same study described comparable reductions in *C. borealis* second-instar larvae collected from imidacloprid- and thiamethoxam-treated plots relative to controls^68^.

Similar to the *P. japonica* results, Renkema et al.^66^ reported the comparative abundance of the pest scarab *Amphimallon majale* Razoumowsky among plots treated with low and high application rates of formulated neonicotinoid turf products. Significantly less third-instar larvae were recovered from imidacloprid treated plots compared to thiamethoxam treated plots. Overall, soil-dwelling grubs of pest scarabs are challenging to scout and display a patchy distribution. Preventative soil and seed neonicotinoid treatments are often favored with the goal of species-specific efficacy to AIs. Our data further support this AI-specific sensitivity under acute and chronic conditions to imidacloprid and thiamethoxam with *Canthon spp.* exhibiting greater sensitivity to imidacloprid under both experimental conditions.

### Detoxification of neonicotinoids

Insects employ a number of compensatory behavioral and physiological mechanisms to survive the adverse effects of insecticide exposure. Detoxifying enzymes, such as cytochrome P450 monooxygenases (CYP450), carboxylesterases, and glutathione *S*-transferases, play an essential role in insecticide metabolism and are actively expressed in pest scarab beetle taxa^69,70^. Of note, the induction of CYP450 represents a critical mechanism of neonicotinoid detoxification^69^. During the first 24 h of the 10-day soil bioassay, qualitative observations of beetles across the neonicotinoid treatments revealed a period of morbidity and intoxicated behaviors of rolling on their backs and twitching of legs. Similar sub-lethal effects from imidacloprid products were described by Zhu et al.^71^, where exposed third-instar European chafer larvae, *A. majale*, were paralyzed or displayed sustained inactivity. Arrestant behavior increases the susceptibility to exogenous biotic and abiotic factors, as well as increases prolonged AI contact. Because of irreversible binding properties by neonicotinoids to insect nAChRs, exposed insects experience cumulative toxicity over time with delayed mortality after a low-dose, chronic exposure^72^.

Here, the *Canthon spp.* that did not succumb to the treatment resumed conventional behavior (i.e., walking upright and burrowing); however, beetles were not individually tracked throughout the study. The assumed recovery of some beetles prompted the addition of acetone rinses to quantify neonicotinoid residues on the external cuticles of exposed beetles at test termination, which yielded no detection among all neonicotinoid treatments. For further context, beetles were fed and active throughout the soil bioassay. Metabolic rate in dung beetles is dynamic and responds to a number of environmental conditions^73–75^, which primarily include body size, foraging activity, and temperature. Previous investigations on the sub-lethal effects of ivermectin exposure on dung beetle physiology reported increased metabolic rates and expression of heat shock proteins^76^, describing a potential response in metabolism from pesticide exposure.

### Routes of neonicotinoid exposure

The life history strategies of taxa belonging to the subfamily Scarabaeinae are almost exclusively tied to dung resources causing species to potentially be exposed to a suite of synthetic chemicals used to suppress livestock pests and parasites^36,44,77^. As a result, dung with pesticide residues has been studied extensively. Ingestion of and development in dung containing veterinary medical products and pyrethroids causes direct larval and adult mortality, and negatively impact body condition and reproductive output^46–49,77^. However, airborne and soil exposures of other compounds have been largely overlooked.

Dung beetle forage flight times and diel patterns are governed by a number of abiotic and biotic factors^43^. The length of time between foraging periods will differ seasonally with temperature and reproductive status with some reported flight times ranging from 10 min to 2 h^42^. Multiple studies have confirmed that vagile insects traversing agricultural fields during the planting of neonicotinoid-treated seeds are exposed to substantial concentrations of particle-bound AIs^19,20^. Tapparo et al.^19^ measured neonicotinoid-bound particulate matter in air > 9 m into the field margins from 200 to 1600 ng/m^3^, which translated to 25–100.5 ng per caged-honeybee. Mortality for imidacloprid-exposed beetles was 100% for all concentrations above 52.8 ng/beetle. Girolami et al.^20^ quantified imidacloprid residues on bees following a field exposure to seed dust and measured 29–3661 ng/bee; the latter value is more than 80 times greater than the imidacloprid LD90 value of 43.3 ng/beetle reported in the present study. In a similar study, Girolami et al.^15^ measured 1221 ng imidacloprid/bee across a transect of 9 m from an unmodified seed driller exhaust pipe, with 199 ng/bee at the 9-m sampling point. Recently, emissions from beef feedlots were found to generate similar concentrations of neonicotinoids from < LOQ to 1125 ng/m^3^ of imidacloprid. Projected emissions amounted to 13.3 × 10^9^ ng of imidacloprid per day^16^, highlighting another potential mode of airborne contact. Reported values originating from both row crop and livestock operations are thus potentially lethal to *Canthon spp*. during field-realistic scenarios. However, the authors acknowledge that abiotic (i.e., temperature, wind, humidity) and morphological (i.e., cuticle thickness, setae density) variables may influence exposure.

Neonicotinoids are frequently detected in soils post-harvest and soils proximal to cultivated areas^32^. Field surveys throughout the United States have reported neonicotinoid concentrations within the bounds of doses used in the present study. For example, mean neonicotinoid concentrations (imidacloprid and clothianidin) from footslope soils sampled in Iowa seasonally ranged from 10 to 28 µg/kg^56^. In Illinois, de Perre et al.^55^ quantified neonicotinoid soil concentrations (clothianidin) and measured approximately 2–4 µg/kg of soil after 200 days. Samples from cultivated field soils in Missouri had a maximum neonicotinoid concentration (clothianidin) of 41.7 µg/kg with field margin soils measuring < 3 µg/kg^12^. Settling of seed dust on non-target soils was reported by Krupke et al.^14^ in field margins proximal to maize and soybean growing operations and measured 2.9–7.3 µg/kg of imidacloprid.

Intentional livestock management practices and unintentional movements of wildlife may deposit dung resources on the surface of neonicotinoid-treated and nearby contaminated soils. An exposure scenario unique to dung beetles involves post-harvest forge systems, where crop residues (e.g., corn and sorghum) are used to feed cattle on cultivated land planted with neonicotinoid-treated seeds^78^. Cattle produce 8–12 dung pats per day^79^, and larger herds will attract greater numbers of dung beetles^80^. Seasonal availability of dung resources through space and time near operations that implement these practices may be luring dung-seeking beetles to soils with higher neonicotinoid concentrations. In some crop systems winter wheat, *Triticum sp*. is grown for grazing and cattle are pastured with wheat beginning one month from planting. This practice is widely used in Kansas and Oklahoma, where planted wheat seeds are typically treated with imidacloprid or thiamethoxam^81^. With the highest neonicotinoid concentrations measured in soil immediately post-seeding^32^, early season grazing may expose dung beetles to much higher levels of active ingredient. For a roller species, like *Canthon spp.*, the beetles will move dung over 10 m across a soil surface before burying the dung ball and repeating the process^82^, increasing potential exposure.

Dung beetles increase soil aeration and porosity by burrowing or reworking soils^44^, where extended exposure to soil-bound pesticides may result from incidental ingestion by handling soil particles with their mouthparts or direct penetration through the cuticle. Soil texture, organic matter, ultra-violet radiation, moisture, temperature, and pH will influence the persistence and bioavailability of neonicotinoid AIs^32,33^, and the use of livestock manure, containing high organic carbon, is linked to increased neonicotinoid adsorption and persistence in soil^83^. After a 10-day exposure to imidacloprid-spiked soil, beetle survivorship substantially decreased with a significant difference from the control treatment and 9 µg/kg imidacloprid treatment. In the present study, mean measured soil concentrations across all neonicotinoid treatments were just 21% of the nominal dose. In combination, the complete homogenization of spiked-soil samples and the conditionally irreversible binding of AI to soil particles could complicate efforts to achieve the target dose. The mean measured soil concentration in the 9 µg/kg imidacloprid treatment was 1.5 ± 0.2 µg/kg, suggesting that *Canthon spp*. may be at risk at low concentrations of imidacloprid in soil.

Previous accounts of adult *Canthon spp.* occupancy in brood chambers have ranged up to 10 days for males and 32 days for females^53^, prompting a conceivable prolonged exposure scenario. The primary species used in this study, *Canthon chalcites*, belongs to the roller guild, where beetles will acquire a small portion of dung, roll it into a ball, move it away, and bury it^41^. Telecoprid dung beetle brood chamber depth is typically less than 6 cm^45,54^. To date, the collection of soil samples for neonicotinoid residue analyses has been generally limited to surface soils (approximately 10–15 cm). In the context of dung beetle dung usage and behavior, the vertical distribution of insecticides in soils is relevant to the risk assessment among guilds. Rollers and dwellers tend to have greater extended interaction with surface soils, creating brood chambers near the surface of the soil and beneath dung pats. Tunnelers will construct deeper brood chambers, relocating dung greater than 50 cm into the soil in some cases^41,45^.

Bioturbation has substantial consequences for the physical maintenance and structure of soils. Tunnel depth is a behavior that varies considerably among dung beetle guild classification and the interaction with soil profile may influence behavior and exposure. For example, Grewal et al.^68^ determined imidacloprid- and thiamethoxam-treated turfgrass disrupted the overwintering behavior and vertical migration of *P. japonica* third-instar larvae; more than 50% of the grubs collected from control plots were below 10 cm in the soil column, compared to 10% of the grubs collected from the neonicotinoid-treated plots. Zhu et al.^71^ described arrestant behavior *A. majale*, where in third-instar were not deterred by and could not remotely detect imidacloprid-treated soil. Macropores in soils created by root channels or subterranean invertebrates can facilitate the vertical movement of water in the soil profile and leaching of neonicotinoids^84^. One recent study from southern China extracted soil columns (0–100 cm) from citrus orchards to assess the vertical distribution of neonicotinoids. Subsoil accumulation of neonicotinoids was correlated with increased organic carbon and porosity and decreased bulk density^85^. Samples collected from 10 to 20-year-old orchards had higher neonicotinoid concentrations deeper in the soil profile with the summed total of the five neonicotinoid AIs measuring greater than 10 µg/kg at 40–50 cm. Leaching studies have recovered measurable imidacloprid concentrations at a depth of 60 cm in sandy soils with low organic matter^86^. Radolinski et al.^87^ detected thiamethoxam at a soil depth of 30–45 cm 33 days post-planting of treated seeds, citing greater transport of AI with fine soil particles. Behavioral bioassays or choice- and no-choice tests among species from different behavioral guilds may better characterize the risks associated with these exposure profiles.

The data presented here underscore that dung beetles may be at risk to exposure pathways not previously addressed in the literature, and the fate and distribution of neonicotinoids in air and soil are of concern. While not the focus of the present study, recent investigations have measured the daily intake of neonicotinoids via livestock feed^50^ and have detected imidacloprid in dung at 2.1 µg/kg^88^ with unknown risks to beneficial coprophagous insects.

## Conclusions

Landscape-level agricultural matrices impact the success of dung beetles, from individual body condition to community diversity and abundance^35–37,39,63^. Worldwide declines among monitored dung beetle populations continue with limited data on the effects of broad-spectrum insecticides, specifically neonicotinoids. Neonicotinoid use and application continues to receive heavy scrutiny with regulatory action ranging from banning or phasing out their use to defining mitigation strategies^89–91^. Our data and other studies on scarab beetles suggest there is a significant difference in toxicity among neonicotinoid AIs, and we provide the potential risks associated with environmentally relevant concentrations under two exposure scenarios.

These data add to the ongoing conversation on responsible neonicotinoid use, their role in integrated pest management, and the cost–benefit of insurance-based pest management approaches, i.e., seed treatments^10^. Dung beetles are beneficial and established biological indicators of pasture condition that are sensitive to contamination^36,44,63^. Beyond lethality from exposure, the addition of sub-lethal (e.g., body condition, reproductive output, movement) and functional (e.g., dung decomposition, seed dispersal, soil structure) endpoints could further highlight the non-target impacts of neonicotinoids on pasture ecosystems. In the U.S., ecological effects test guidelines for pesticide risk assessment and registration are mostly limited to pollinators^92,93^ with other beneficial insects considered on a case-by-case basis^94^, representing a narrow characterization of the potential risks to terrestrial insects^12^. Beetles are among the most diverse and abundant arthropods, sensitive to organic and inorganic pollution, and informative bioindicators that merit more attention^95^.

## Data Availability

The datasets used and/or analyzed during the current study available from the corresponding author on reasonable request.
